# Unveiling the Regulatory Mechanism of Tibetan Pigs Adipogenesis Mediated by WNT16: From Differential Phenotypes to the Application of Multi-Omics Approaches

**DOI:** 10.3390/ani15131904

**Published:** 2025-06-27

**Authors:** Qiuyan Huang, Kunli Zhang, Fanming Meng, Sen Lin, Chun Hong, Xinming Li, Baohong Li, Jie Wu, Haiyun Xin, Chuanhuo Hu, Xiangxing Zhu, Dongsheng Tang, Yangli Pei, Sutian Wang

**Affiliations:** 1 State Key Laboratory of Swine and Poultry Breeding Industry, Guangdong Provincial Key Laboratory of Animal Breeding and Nutrition, Institute of Animal Science, Guangdong Academy of Agricultural Sciences, Guangzhou 510640, China; huangqqiu2022@163.com (Q.H.); mengfanming@gdaas.cn (F.M.); hongchun202210@163.com (C.H.); lxm980421@163.com (X.L.); boohom@163.com (B.L.); jiewu_email@163.com (J.W.); xinhaiyun503@163.com (H.X.); 2 Key Laboratory of Livestock Disease Prevention of Guangdong Province, Guangdong Provincial Observation and Research Station for Animal Disease, Institute of Animal Health, Guangdong Academy of Agricultural Sciences, Guangzhou 510640, China; 3 Sericultural & Agri-Food Research Institute, Guangdong Academy of Agricultural Sciences, Guangzhou 510640, China; linsen@gdaas.cn; 4 Guangxi Key Laboratory of Animal Breeding, Disease Control and Prevention, College of Animal Science and Technology, Guangxi University, Nanning 530004, China; hch64815@gxu.edu.cn; 5 School of Medicine, Foshan University, Foshan 528051, China; zhu_xiangxing@126.com (X.Z.); tangdsh@163.com (D.T.); 6 Guangdong Provincial Key Laboratory of Animal Molecular Design and Precise Breeding, Key Laboratory of Animal Molecular Design and Precise Breeding of Guangdong Higher Education Institutes, School of Life Science and Engineering, Foshan University, Foshan 528225, China; peiyangli@163.com

**Keywords:** pigs, IMF, transcriptome, metabolome, fat deposition

## Abstract

The aim of this study is to investigate the physiological characteristics and regulatory mechanisms of porcine intramuscular fat (IMF), subcutaneous fat (take back fat (BF), for example), and visceral fat (take perienteric fat (PF), for example) to address the challenge of optimizing meat quality without excessive fat deposition. Many improved breed pigs have fast growth rates, high lean meat rates, and low subcutaneous fat deposits, but they also have low IMF content, resulting in poor meat quality. There is usually a positive correlation between intramuscular fat and subcutaneous fat deposits. This study selected eight-month-old female Tibetan pigs as experimental subjects. After slaughter, fat samples were collected. Histological differences in adipocyte morphology were observed via hematoxylin–eosin (HE) staining of tissue sections, and phenotypic characteristics of different adipose tissues were analyzed through fatty acid composition determination. Transcriptome sequencing and untargeted metabolomics were employed to perform pairwise comparisons between different fatty tissues to identify differentially expressed genes and metabolites. A siRNA interference model was constructed and combined with Oil Red O staining and lipid droplet optical density measurement to investigate the regulatory role of WNT16 in adipocyte differentiation. Comparative analysis of phenotypic and fatty acid composition differences in adipocytes from different locations revealed that IMF adipocytes have significantly smaller areas and diameters compared to other fat depots and contain higher levels of monounsaturated fatty acids. Integrated transcriptomic and metabolomic analyses identified differential expression of WNT16 and L-tyrosine, both of which are involved in the melanogenesis pathway. Functional validation showed that inhibiting WNT16 in porcine preadipocytes downregulated adipogenic regulators and reduced lipid droplet accumulation. This cross-level regulatory mechanism of “phenotype detection–multi-omics analysis–gene function research” highlighted WNT16 as a potential key regulator of site-specific fat deposition, providing new molecular targets for optimizing meat quality through nutritional regulation and genetic modification.

## Simple Summary

This study unveils the regulatory role of WNT16 in adipogenesis in Tibetan pigs through phenotypic detection, multi-omics analysis, and gene function validation. The research found that intramuscular fat cells have significantly smaller areas and diameters compared to other fat types and contain higher levels of monounsaturated fatty acids. Transcriptomic and metabolomic analyses identified differential expression of WNT16 and L-tyrosine, both involved in the melanogenesis pathway. Functional validation showed that inhibiting WNT16 reduces lipid droplet accumulation and downregulates adipogenic regulators. This study provides new targets for optimizing meat quality, which is significant for the sustainable development of the pig industry and improving pork quality.

## 1. Introduction

Pork quality is a complex trait involving multiple factors, among which muscle fiber type, fat content and distribution, and fatty acid composition are closely related to meat tenderness, juiciness, and flavor. Among the many factors that influence pork quality, the characteristics of fat content play a key role [1]. Pig fat is mainly classified into intramuscular fat (IMF), subcutaneous fat (including back fat and abdominal fat), and visceral fat (perienteric fat, perihepatic fat, and perirenal fat), according to the deposition site [2]. Among them, the IMF mainly affects the quality of pork.

In production, IMF in pigs is formed by hypertrophy and hyperplasia of intramuscular adipocytes during the late developmental stage [1]. IMF plays an extremely crucial role in lipid metabolism and the formation of meat flavor. An appropriate increase in IMF can significantly improve meat quality by enhancing tenderness, juiciness, and flavor. The reality, however, is that when we aim for high intramuscular fat content, the level of fat deposition in other parts of the pig also increases significantly. The excessive depositions of subcutaneous fat and visceral fat reduce feed utilization and increase breeding costs [3]. In addition, the deposition of visceral fat is closely related to physiological health. It participates in whole-body energy metabolism and fat regulation, and excessive accumulation of visceral fat may induce a series of metabolic diseases [4], such as insulin resistance and cardiovascular diseases. Paying attention to the distribution of fat in the study of fat deposition regulation helps us to comprehensively understand the regulatory mechanism of fat metabolism from the perspective of overall health, providing theoretical support for ensuring the efficient breeding of pigs and a healthy diet for humans. Our study aims to explore regulatory factors that can increase IMF deposition without increasing or even reducing the deposition of subcutaneous fat (take back fat, for example) and visceral fat (take perienteric fat, for example) and precisely improve meat quality traits. The related findings are expected to provide a more scientific regulatory strategy for pig breeding, enabling more efficient and market-compliant breeding production while ensuring meat quality.

Here, it is worth mentioning that genetic selection of pigs has helped to improve fattening traits [5]. Duroc, Landrace, and Yorkshire pigs are known for their fast growth, high feed conversion, and lean meat content [6,7]. Jinhua, Laiwu, and Min pigs are famous for their high fat content, including abundant marbling and unsaturated fatty acids, and their high-quality meat [8,9,10]. Tibetan pig (TP) is a local pig breed with unique genetic and biological characteristics. It has long lived in special environments. Through long-term natural selection and adaptation, it may have developed many characteristics in the regulatory mechanisms of energy metabolism, fat deposition, and stress resistance that distinguish it from other pig breeds [11]. Some studies have identified genes like *PHACTR1*, *SFI1*, and *EPM2A* that can enhance the cardiopulmonary function and fat metabolism of TPs living in high-altitude areas [11,12]. Under cold stress, triglycerides in subcutaneous fat are decomposed, the content of saturated fatty acids decreases, lipid peroxidation in visceral fat is reduced, and the contents of fatty acids, such as C12:0, C14:0, and C24:0, are significantly decreased, thereby influencing fat deposition [13]. We have bred a group of TPs adapted to low-altitude areas. Studying their fat differences helps reveal fat deposition patterns in different body parts and the genes and molecules behind high-quality meat formation.

In recent years, a series of advances have been made in the study of fat deposition in pigs using multi-omics analysis. Yan investigated the impact of different temperature environments on pig fat. It was found that under cold stress, triglycerides in subcutaneous fat were decomposed, and oxidative stress and lipid peroxidation in visceral fat were reduced. The contents of stearic acid (C18:0) in subcutaneous fat and C12:0, C14:0, and C24:0 in visceral fat were significantly decreased. The levels of γ-linolenic acid (C18:3n6) and arachidonic acid (C20:4n6) in both adipose tissues showed a downward trend. Cold stress also decreased the total saturated fatty acid content in subcutaneous fat and affected the content of individual fatty acids in visceral fat [14]. By comparing Jinhua pigs in the high-fat group and the low-fat group, the results showed that the expression levels of fat deposition-related genes, such as FAS, LPL, and PPARγ, in the abdominal fat of the high-fat group were significantly higher than those in the low-fat group of Jinhua pigs [13]. Peng et al. selected Tibetan pigs and Yorkshire pigs, which have differences in obesity and fatty acid composition, as research objects. RNA-Seq analysis revealed that the genes FASN, ACACA, and SCD play regulatory roles in affecting the level of monounsaturated fatty acids and may be involved in the regulation of fat deposition [15].

Our research conducted a comprehensive and in-depth study on the content, microstructural characterization, and lipidomic profiling of IMF, back fat (BF), and perienteric fat (PF) in TPs. Through this systematic research strategy, we analyzed the fats in different parts of TPs, identified differentially expressed genes and metabolites enriched in different parts, and verified the functions of key genes regulating fat deposition in TPs at the cellular level. From the phenotype to cellular mechanism of gene action, our study is based on a multi-omics approach, combined with histological analysis and gene function verification, to explore the regulatory mechanism of fat deposition traits. We aim to identify core genomic loci and signaling networks that affect the fat content of pigs, thereby elucidating the biological processes determining the formation of ideal meat quality.

## 2. Materials and Methods

### 2.1. Animal Ethics Statement

This research strictly adhered to the bioethical guidelines validated by the Guangdong Academy of Agricultural Sciences and the Animal Welfare Committee (Ethical Permit ID: 2022070).

### 2.2. Experimental Design

This study centered on porcine fat characteristics and regulatory mechanisms, designating specific experimental groups for analysis. Researchers selected 8-month-old female Tibetan pigs as their experimental subjects. After slaughter, they collected samples of IMF, BF, and PF. For comparison, they observed differences in adipocyte morphology across these fat types via HE staining and analyzed phenotypic traits by determining fatty acid compositions. Transcriptome sequencing and metabolomics were employed for pairwise comparisons between the different fat tissues to identify differentially expressed genes and metabolites. A siRNA interference model was also constructed to explore WNT16′s role in adipocyte differentiation, alongside Oil Red O staining and lipid droplet optical density measurements. The comparisons mainly focused on the phenotypic, fatty acid, gene expression, and metabolic differences between IMF, BF, and PF to understand their distinct characteristics and regulatory mechanisms.

### 2.3. Animals and Sample Preparation

TPs were raised under the same conditions at the Institute of Animal Science, Guangdong Academy of Agricultural Sciences. The indoor temperature and lighting were in a natural state without control. The formulation of experimental diets used corn and soybean meal as the main raw materials. For different growth stages, the levels of crude protein, trace minerals, and vitamin contents were adjusted to meet the guidelines established by the National Research Council. To study the effects of fats from different parts of TPs on growth performance, carcass performance, and meat flavor, 3 eight-month-old female TPs were selected at the transcriptome level, and 6 eight-month-old female TPs were selected at the non-targeted metabolomics level. Subsequently, these pigs were all electrocuted and slaughtered in accordance with national slaughter standards. Five samples of IMF, BF, and PF were collected, respectively. One of the samples was preserved in 4% paraformaldehyde for the preparation of tissue sections, and the remaining four samples were stored frozen at −80 °C for subsequent fatty acid determination, transcriptome analysis, metabolomics analysis, and detection of related indicators.

### 2.4. Isolation and Culture of Preadipocytes

In this study, 7 days of TPs were obtained from the Animal Science Institute of Guangdong Academy of Agricultural Sciences and euthanized by exsanguination. After disinfection with 75% ethanol, inguinal and dorsal adipose tissues were excised under sterile conditions, taking care to avoid contamination and the inclusion of muscle tissues. The adipose tissues were placed in a centrifuge tube in PBS solution containing 3% penicillin-streptomycin. The tissues were immersed in ethanol for 1 min, followed by rinsing to remove ethanol and debris. The minced adipose tissues were then digested with 1 mg/mL of Type I collagenase at 37 °C for 1 h with agitation every 10 min. The digested tissues were sequentially passed through 70 μm and 40 μm cell meshes, Spun at 800 g multiple times, and washed with DMEM/F12 + 10% FBS + 1% antibiotic-antimycotic (called complete medium). The cells were resuspended in complete medium and incubated at 37 °C in a 5% CO_2_ humidified atmosphere.

### 2.5. The Preparation of Fat Tissue Slices and Measurement of Cells

IMF, BF, and PF were sampled, the samples were sent to Jinzhi Testing Technology Co., Ltd. (Guangzhou, China)., fixed in 4% paraformaldehyde, dehydrated, made transparent, embedded in paraffin, sectioned, dewaxed, rehydrated, and stained with H&E staining. Sections were imaged at 100× and 400×. The scale units on the ruler were calibrated using ImageJ software 1.51. At least 30 adipocytes were selected from the field of view of each section, and we used ImageJ to measure their respective areas and diameters.

### 2.6. Determination of Fatty Acids

The fatty acid content was detected in accordance with the national standard GB 5009.168-2016 [16]. The experimental procedures were as follows: 150 mg of IMF, BF, and PF were, respectively, weighed and placed into beakers. After a series of processes, including hydrolysis, condensation, saponification, and fatty acid methylation, a capillary chromatographic column with a length of 100 m, an inner diameter of 0.25 mm, and a film thickness of 0.2 µm was adopted. The injector temperature was set at 270 °C, and the detector temperature was set at 280 °C. The temperature programming was carried out as follows: maintaining at 100 °C for 13 min, heating from 100 °C to 180 °C at a rate of 10 °C/min, then heating at a rate of 1 °C/min for 20 min, and subsequently heating at a rate of 4 °C/min and maintaining for 10.5 min. Nitrogen was used as the carrier gas, and the split ratio was 100:1 for chromatographic analysis.

### 2.7. Transcriptome Sequencing

The RNA extraction and sequencing were conducted by Majorbio Bio-Pharm Technology Co., Ltd. (Shanghai, China) on the Illumina NovaSeq X Plus platform (PE150). After RNA extraction from the test samples, the Agilent 2100 detection profile showed that each sample had a high peak concentration, and all RIN values were ≥7. Following quality control using fastp, the sequencing data were mapped to the Sscrofa11.1 reference genome with HISAT2. Gene expression was quantified using RSEM, and differential expression analysis was performed with DESeq2 (Version 1.24.0, http://bioconductor.org/packages/stats/bioc/DESeq2/, accessed on 17 November 2022), identifying differentially expressed genes (DEGs) based on |log2FC| ≥ 1 and FDR < 0.05. Functional enrichment analysis of DEGs was carried out using the GO and KEGG databases.

### 2.8. Untargeted Metabolite Assay

The untargeted metabolomics analysis was conducted by Majorbio Bio-Pharm Technology Co., Ltd. (Shanghai, China) using the UHPLC-QExactiveHF-X (Thermo, Waltham, MA, USA) system for LC-MS/MS. Quality control samples were inserted at regular intervals to ensure stability and reproducibility. Data were processed by removing variables with RSD > 30% and log10-transforming the remaining data. Metabolites were identified by comparing MS and MS/MS spectra with HMDB, METLIN, and an in-house database. Differentially expressed metabolites (DEMs) were identified using the ropls R package (v1.6.2) based on VIP > 1 and *p* < 0.05. The pathways involved were annotated using KEGG, and pathway enrichment analysis was performed using scipy (Python v1.0.0).

### 2.9. Combined Transcriptome and Metabolome Analysis

The correlation analysis between the obtained DEGs and DEMs was conducted using the scipy library of Python (V1.0.0). The expression relationships between DEGs and DEMs were further analyzed using OmicsPLS (V2.0.2) and vegan (V2.6.4). Subsequently, enrichment analysis was conducted by mapping DEGs and DEMs to the KEGG pathway database. Based on *p* < 0.05, pathways significantly enriched with genes and metabolites were identified. Data analysis was completed with the assistance of the Maji Cloud platform (cloud.majorbio.com, accessed on 17 November 2022).

### 2.10. qRT-PCR Analysis

Total RNA from animals was extracted using a total RNA extraction kit (Tiangen, Beijing, China). The concentration of RNA was measured using an ultraviolet spectrophotometer (Implen, München, Germany), and the 260/230 ratio was detected to evaluate the sample quality.

Subsequently, cDNA was generated using the HiScript III All-in-one RT SuperMix (Vazyme, Nanjing, China) for qPCR. Reverse transcription was carried out at 50 °C for 15 min and 85 °C for 15 s, with the reaction mixture prepared on ice. The synthesized cDNA was stored at −20 °C. Real-time quantitative PCR was performed using the CFX96 real-time system and Taq Pro Universal SYBR qPCR Master Mix (Vazyme, Nanjing, China). Primers were designed using Primer6.0 (Table 1). Gene expression was analyzed using cDNA as the template and β-actin as the internal control, with three biological and technical replicates for each reaction. The reaction volume of qPCR was 20 μL, consisting of 10 µL of 2× Taq Pro Universal SYBR qPCR Master Mix, 1 µL of cDNA template, 1 µL each of upstream and downstream primer, and 7 µL of water.

The PCR amplification procedure included initial denaturation at 95 °C for 30 s, followed by 40 cycles of 95 °C for 10 s and 60 °C for 30 s. The melting curve was obtained using the instrument’s default settings, and relative gene expression levels were determined using the 2^−ΔΔct^ method.

### 2.11. siRNA Interference and Adipogenic Differentiation

When the cell confluence reached 100%, the cells were seeded into a 6-well plate at a density of 4 × 10^4^ cells per well. When the cell confluence reached 80%, the medium was replaced with antibiotic-free basic culture medium (DMEM + 10% FBS). Subsequently, transfection was performed using Lipofectamine™ 3000 transfection reagent (Invitrogen, Carlsbad, CA, USA). Each siRNA was designed with three replicates (Table 2). The transfection system was as follows: Solution A: 125 µL Opti-MEM culture medium + 5 µL Lipofectamine™ 3000; Solution B: 125 µL Opti-MEM culture medium + 2.5 µg siRNA. After mixing Solution A and Solution B, the mixture was incubated for 15 min and then added to the cell culture medium. Six hours after transfection, the basic culture medium was replaced. The interference efficiency of si-WNT16 was verified using qRT-PCR technology. For the adipogenic differentiation experiment, 24 h after siWNT16 transfection, the cells were subjected to differentiation induction by replacing the standard culture medium with an adipogenic induction medium (DMEM containing 10% FBS, 0.5 mM 3-isobutyl-1methylxanthine, 1 µM dexamethasone, and 10 μg/mL insulin; Sigma, Los Angeles, CA, USA) and cultured for 2 days. The differentiation medium was then replaced with a maintenance medium (DMEM containing 10% FBS and 10 μg/mL insulin) and cultured for 6 days. After the induction was completed, the medium was discarded, the cells were washed, fixed with Oil Red O for 30 min, and then stained and rinsed. Finally, 50 μL of the treated isopropanol was taken from the cells and added to 4 wells of a 96-well plate, and the absorbance (OD value) was measured at 490 nm.

### 2.12. Statistical Analysis

All experimental data were analyzed using Excel 2013. qRT-PCR and adipocyte measurement data were analyzed via one-way ANOVA or independent samples *t*-tests using GraphPad Prism 10 (GraphPad Software Inc., San Diego, CA, USA). Fatty acid data were compared using one-way ANOVA in SPSS 20. Results are presented in the text as mean ± standard error, with *p* < 0.05 indicating statistical significance.

## 3. Results

### 3.1. Comparative Analysis of Cell Morphology of Adipose from Different Sites

**Figure 1:**
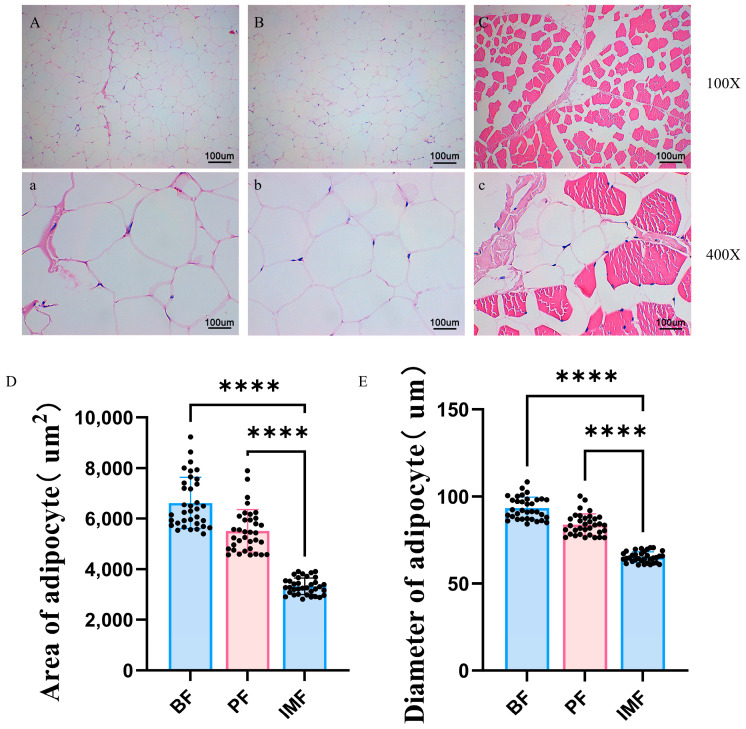
Hematoxylin–eosin (HE) staining of adipocytes in TPs. (**A**,**a**) Images of the BF tissue sections of TPs under 100× and 400× magnifications, respectively. (**B**,**b**) Images of the PF tissue sections of TPs under 100× and 400× magnifications, respectively. (**C**,**c**) Images of the IMF tissue sections of TPs under 100× and 400× magnifications, respectively. (**D**) The ratio of fat area. (**E**) The ratio of fat diameter. The asterisk (****) denotes a statistical difference between groups (*p* < 0.05). The sample size N ≥ 3.

Fat distribution, IMF content, and fat deposition are crucial determinants of pork quality. Notably, significant differences in fat deposition patterns exist across various anatomical sites among different pig breeds. In this study, we focused on dissecting the histological characteristics of the IMF, BF, and PF of TPs. Figure 1 presents the histological comparisons of the adipose tissues in TPs. During the experimental procedures, tissue sections of the three adipose regions from TPs were carefully observed under 100× and 400× magnifications. For the 100×-magnified sections, the areas of adipocytes were precisely measured, and the diameters were calculated based on the measured areas. The results showed that BF adipocytes exhibited irregular, rounded, and densely packed morphologies with relatively large volumes. The adipocytes in the PF tissue were similar to those in the BF, sharing similar irregular, rounded, and large-volume characteristics. In contrast, the IMF adipocytes were smaller in size, showed heterogeneous dimensions, and predominantly resided in the intercellular spaces of muscle cells. Comparative analyses of the areas and diameters of adipocytes from different adipose sites revealed that the area of IMF adipocytes in TPs was significantly smaller than that of both BF and PF adipocytes (*p* < 0.05). Similarly, the diameter of IMF adipocytes was also notably less than that of adipocytes at the other two sites (*p* < 0.05).

### 3.2. Fatty Acid Analysis of Adipose Tissue from Different Sites

**Figure 2:**
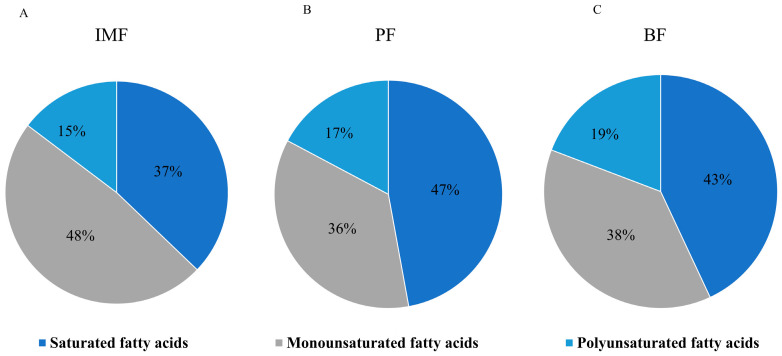
Percentage of fatty acids in different parts of TPs. (**A**) Percentage of fatty acid composition in IMF, (**B**) Percentage of fatty acid composition in PF, (**C**) Percentage of fatty acid composition in BF.

In this study, the fatty acid contents of IMF, BF, and PF of TPs were measured. A total of 25 fatty acids were detected in the IMF, BF, and PF of TPs (Table 3). Among them, there were 10 saturated fatty acids, such as C8:0, C16:0, C18:0, and C22:0. The content of C16:0 was the highest among the three sites, followed by C18:0. There were five monounsaturated fatty acids, and the content of C18:1 (cis-9) accounted for more than 32% of the total fatty acid composition. There were 10 polyunsaturated fatty acids in total, with C18:2 (all-cis-9, 12) having the highest content. According to the pie chart, the proportions of saturated fatty acids in IMF, BF, and PF were 37%, 47%, and 43%, respectively, and the contents of saturated fatty acids in BF and PF were higher than those in IMF. The proportions of monounsaturated fatty acids were 38%, 36%, and 48%, respectively, and the content of monounsaturated fatty acids in IMF was higher than that in BF and PF. The proportions of polyunsaturated fatty acids in IMF, BF, and PF were 15%, 17%, and 19%, respectively, and the differences in the contents of unsaturated fatty acids among the three sites were relatively small (Figure 2A–C).

### 3.3. Transcriptome Analysis of Adipose Tissue from Different Sites

**Figure 3:**
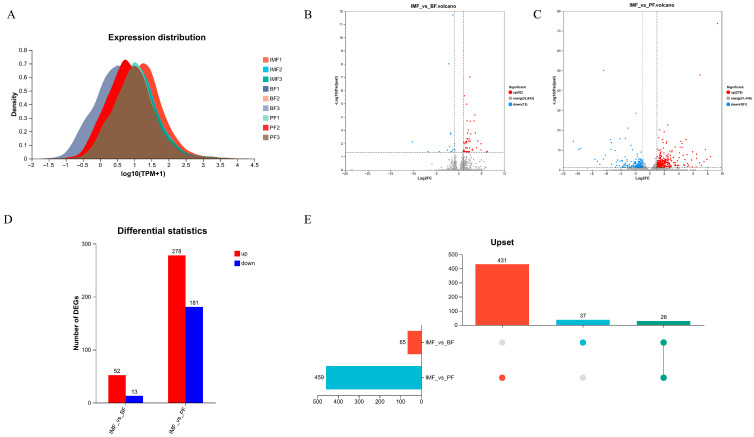
Number of differentially expressed mRNAs in TPs in IMF, BF, and PF. (**A**) Expression distribution of samples, (**B**) volcano plot of IMF vs. BF, (**C**) volcano plot of IMF vs. PF, (**D**) statistical table of differential genes between groups, and (**E**) gene set upset diagram analysis.

The findings demonstrated that the expression levels across samples in the TP transcriptome were effectively illustrated using density plots (Figure 3A). The density curves for each sample exhibited high and concentrated peaks, which were approximately symmetrical. This indicates that the expression levels of the majority of genes were relatively uniform, and the data distribution was consistent without evident skewness. The good concordance among the samples suggests that the data were reliable and suitable for further experimental analyses. The threshold was set as (|log_2_FC|) ≥ 1 and *p* ≤ 0.05. During the differential expression analysis of TP samples, a comparison between IMF and BF led to the identification of 65 differential genes. Among these, 52 genes were upregulated, while 13 genes were downregulated (note that the data for IMF vs. BF were reported previously [17]). When comparing IMF with PF, a total of 459 differential genes were detected, with 278 genes showing upregulation and 181 genes showing downregulation (Figure 3B–D). In the comprehensive comparison among the 3 types of fat, a total of 28 common differentially expressed genes were successfully identified (Figure 3E).

**Figure 4:**
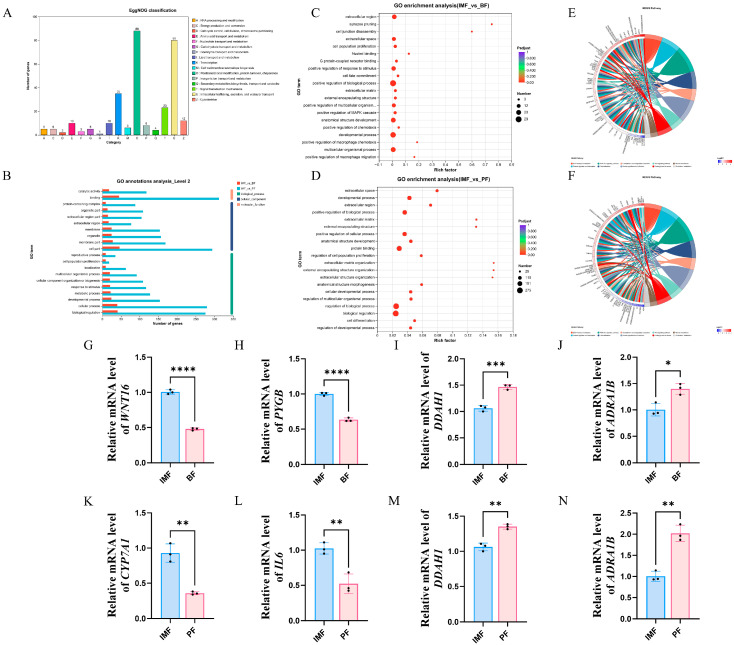
Transcriptomic differential gene enrichment analysis. (**A**) EggNOG classification statistics between groups. (**B**) GO annotations analysis of IMF vs. BF and IMF vs. PF. (**C**) GO enrichment analysis of IMF vs. BF. (**D**) GO enrichment analysis of IMF vs. PF. (**E**) KEGG enrichment chord diagrams of IMF vs. BF. (**F**) KEGG enrichment chord diagrams of IMF vs. PF. (**G**–**J**) qRT-PCR of the expression of *WNT16*, *PYGB*, *DDAH1*, and *ADRA1B* in IMF vs. BF. (**K**–**N**) qRT-PCR of the expression of *CYP7A1*, *IL6*, *DDAH1*, and *ADRA1B* in IMF vs. PF (* *p* < 0.05, ** *p* < 0.01, *** *p* < 0.001, and **** *p* < 0.0001).

Subsequently, functional annotation analysis was performed on these differential genes. In the analysis of TPs, the EggNOG annotation analysis revealed a total of 16 functional types (Figure 4A). Common functional categories, such as energy production and conversion, amino acid transport and metabolism, nucleotide transport and metabolism, lipid transport and metabolism, inorganic ion transport and metabolism, and secondary metabolites biosynthesis, transport, and catabolism, were annotated among them. These pathways might be associated with lipid deposition in fat and meat flavor. Further, GO pathway annotation was performed for IMF vs. BF and IMF vs. PF (Figure 4B). Then, under the premise of *p* < 1, the top 20 GO pathways’ enrichment analysis results were displayed (Figure 4C,D). Among them, the PI3K-Akt signaling pathway, WNT signaling pathway, and cytokine–cytokine receptor interaction pathway might be significantly enriched in pathways related to fat deposition in TPs.

Additionally, under the condition of *p* < 1, the enriched genes were visually displayed. In each pathway, the top 10 significant DEGs were selected for plotting (Figure 4E,F). The significantly enriched DEGs are located on the left side, while the corresponding pathways are on the right. We mainly focused on genes enriched in signaling pathways that might be related to lipid metabolisms, such as *WNT16*, *DDAH1*, *GPR35*, *ADRA1B*, *TCF7*, *ENPP6*, *ACSM3*, *PYGB*, *IL6*, *SLC6A2*, and *CYP7A1*. For the comparisons of IMF vs. BF and IMF vs. PF, two upregulated and two downregulated DEGs were chosen for qRT-PCR validation. The qRT-PCR results matched the sequencing data (Figure 4G–N), verifying the accuracy of the sequencing.

### 3.4. Metabolome Analysis of Adipose Tissue from Different Sites

The processes of adipose tissue differentiation, growth, and deposition are under the regulation of metabolic pathways. Variations in adipocyte size and fatty acid content have been observed in the IMF, BF, and PF of TPs. To investigate these differences, non-targeted metabolomics techniques were employed to detect small-molecule metabolites within adipose tissues, with the aim of identifying differential metabolites and corresponding regulatory metabolic pathways.

**Figure 5:**
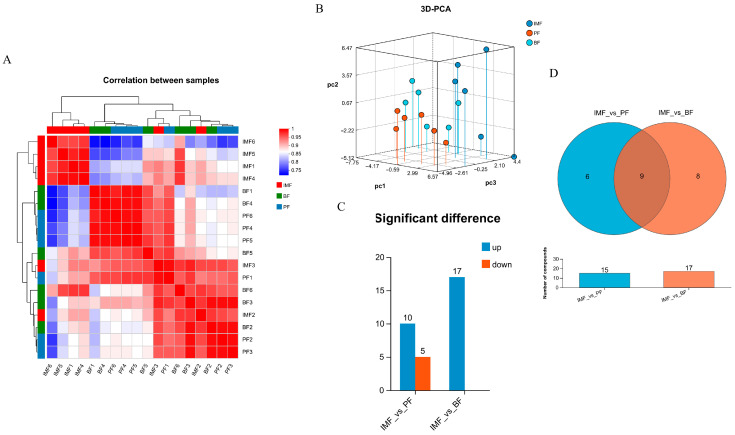
Sample correlation and differential analysis of metabolome. (**A**) Heatmap of correlations among samples, (**B**) 3D-PCA score plots, (**C**) statistical chart of differential metabolite expression levels between groups, and (**D**) Venn analysis of metabolite sets between groups.

The sample correlation heatmap and 3D-PCA revealed the degree of correlation among different samples of TPs as well as the overall differences within groups, facilitating quality control (Figure 5A,B). In TPs, when comparing IMF with BF, a total of 17 metabolites were screened, all of which were upregulated, including L-glutamine, L-tyrosine, Inosine, 2-hydroxycinnamic acid, etc., and no downregulated metabolites were detected. For the comparison between IMF and PF, a total of 15 metabolites were screened, among which 10 metabolites were upregulated, such as creatine, L-glutamine, inosine, etc., and 5 metabolites were downregulated, including LysoPC (17:0), phosphocholine, 13-hydroxy-9-methoxy-10-oxo-11-octadecenoic acid, etc. (Figure 5C). A total of nine common differential metabolites were screened from these three types of fat, namely, LysoPC (17:0), L-carnitine, 3-guanidinopropanoate, L-tyrosine, LysoPC (18:0), and so on (Figure 5D).

**Figure 6:**
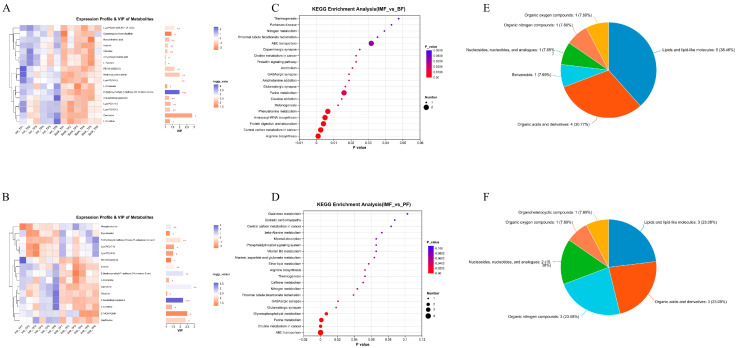
Differential metabolomic analysis. (**A**) VIP analysis outcomes of metabolites of IMF vs. BF. (**B**) VIP analysis outcomes of metabolites of IMF vs. PF. (**C**) KEGG enrichment analyses of metabolites of IMF vs. BF. (**D**) KEGG enrichment analyses of metabolites of IMF vs. PF. (**E**) HMDB compound classifications of metabolites of IMF vs. BF. (**F**) HMDB compound classifications of metabolites of IMF vs. PF (* *p* < 0.05, ** *p* < 0.01, *** *p* < 0.001).

In the metabolite set analysis, OPLS-DA/PLS-DA was employed as the supervised model. The top 30 enriched metabolites with VIP > 1 were visualized through clustering heatmaps and bar charts. Among them, L-glutamine, L-tyrosine, inosine, 2-hydroxycinnamic acid, LysoPC (17:0), LysoPC (16:0), and hydroxybuprenorphine exhibited significant differences (*p* < 0.05; Figure 6A,B).

To identify the functional pathways in which the differential metabolites were involved, KEGG database enrichment analysis was utilized to classify the functional pathways of the metabolite sets of pairwise combinations of the three types of fat in TPs, namely, IMF, BF, and PF, and finally, the enriched pathways and the number of pathway metabolites were obtained. According to the KEGG metabolic pathway classification, differential metabolites were largely found in six main categories, including metabolism, genetic information processing, environmental information processing, cellular processes, organismal systems, and human diseases. Using the secondary classification criteria of the KEGG database, these metabolites were mainly grouped into lipid metabolism, amino acid metabolism, nucleotide metabolism, membrane transport, signal transduction, carbohydrate metabolism, and metabolism of other amino acids (Figure 6C,D). By comparing with the HMDB database, the compound types of TPs were identified as mainly organic acids and derivatives, and lipids and lipid-like molecules.

### 3.5. Transcriptome–Metabolome Combined Analysis

**Figure 7:**
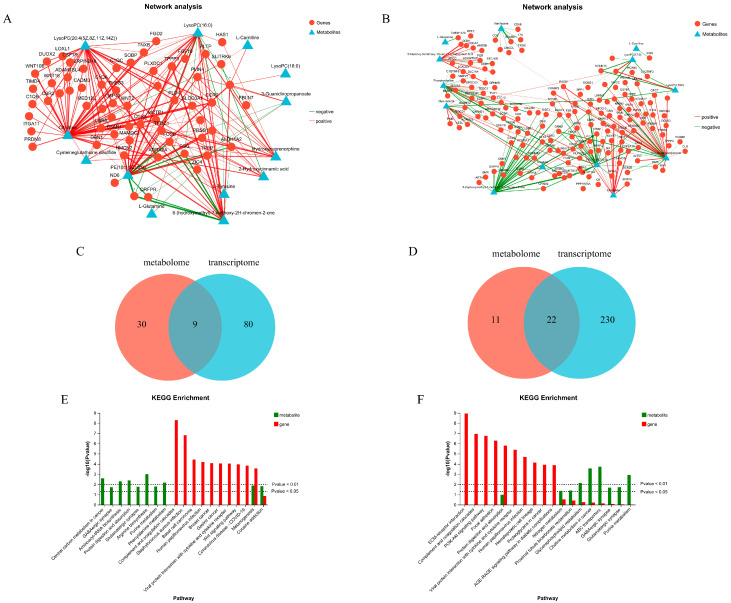
Transcriptomic and metabolomic integrated analysis. (**A**,**B**) Correlation network analysis of IMF, BF, and PF. (**C**,**D**) Venn diagram of KEGG pathway annotation analysis of IMF, BF, and PF. (**E**,**F**) Bar chart of KEGG pathway enrichment analysis for IMF, BF, and PF (figures (**A**,**C**,**E**): IMF vs. BF; figures (**B**,**D**,**F**): IMF vs. PF).

To further explore the key factors affecting pig fat growth and metabolism, this study conducted a combined analysis of the differential gene sets and differential metabolite sets in the fat of three parts of TPs based on transcriptomics and metabolomics. Firstly, the intermolecular interaction relationships were displayed using a correlation network graph calculated from the correlation coefficients between metabolites and genes (Figure 7A,B). Furthermore, in comparison with the KEGG pathway database, 9 common pathways between IMF and BF and 22 common pathways between IMF and PF were annotated (Figure 7C,D). Additionally, KEGG enrichment analysis was conducted under the condition of *p* < 0.05. In the comparison between IMF and BF, differentially expressed genes, including *WNT10B*, *WNT16*, *TSPAN6*, and *WNT2*, were identified, and L-tyrosine was identified as a common differential metabolite. These differential genes and metabolites were all enriched in the melanogenesis pathway. In the comparison between IMF and PF, no relevant metabolites or genes were enriched, which may be due to different metabolic regulatory mechanisms and requires further exploration (Figure 7E,F).

### 3.6. Effect of WNT16 on the Differentiation of Porcine Preadipocytes

**Figure 8:**
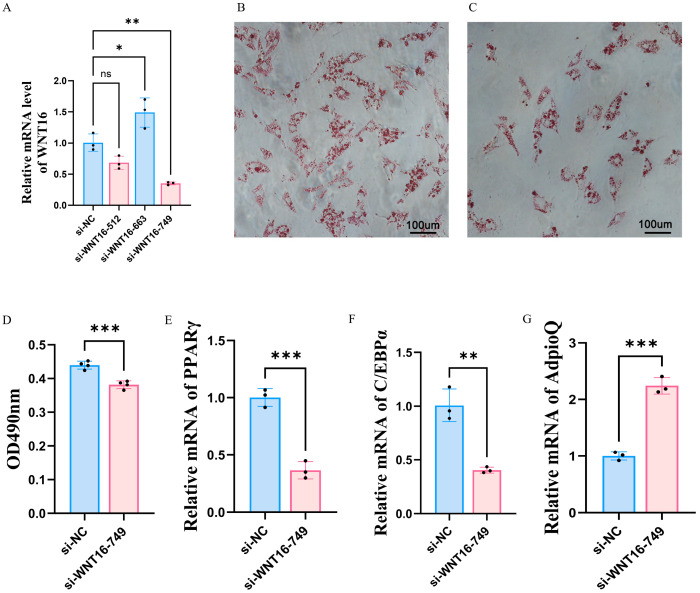
Induction of adipogenic differentiation in adipocytes by *WNT16*. (**A**) Expression of si-WNT16. (**B**) Oil Red staining of si-NC. (**C**) Oil Red staining of si-WNT16-749. (**D**) Determination of OD values after adipogenic induction. (**E**–**G**) qRT-PCR verification of marker genes *PPARγ*, *C/EBPα*, and *AdipoQ* (ns > 0.05, * *p* < 0.05, ** *p* < 0.01, *** *p* < 0.001).

Based on the results of the combined analysis of transcriptomics and metabolomics, the gene with the largest fold change, *WNT16*, was selected as a candidate gene associated with lipid metabolism. To verify the effect of the *WNT16* gene on the adipogenic differentiation process, *WNT16* gene silencing was first carried out. Three pairs of siRNAs were carefully designed for the *WNT16* gene. After transfecting these siRNAs into cells, cell samples were collected for fluorescence quantitative analysis. The results showed that si-WNT16-749 had the best silencing effect. Therefore, si-WNT16-749 was selected for subsequent interference and silencing operations on TPs preadipocytes (*p* < 0.05; Figure 8A). Si-WNT16-749 was transfected into preadipocytes of TPs, and then adipogenic induction and differentiation treatments were performed. On the eighth day of adipogenic differentiation, cell samples were collected for Oil Red O staining. In this study, after the transfection of si-WNT16-749, significant changes in cell phenotype were observed. The number of lipid droplets induced by si-WNT16-749 was significantly lower than that induced by si-NC (Figure 8B,C). Correspondingly, the OD value also decreased (*p* < 0.05; Figure 8D).

At the gene expression level, compared with the si-NC group, the mRNA expression levels of the fat marker genes *PPARγ* and *C/EBPα* showed a significant downregulation trend, while the mRNA expression level of *AdipoQ* was significantly upregulated (*p* < 0.05). This phenomenon further convincingly demonstrated that the deletion of the *WNT16* gene disrupted the metabolic program of adipocytes (Figure 8E–G).

## 4. Discussion

From a histological perspective, the differences in the size and distribution of IMF, BF, and PF cells in TPs may reflect their functional differentiations. The diameter and area of IMF cells in TPs are significantly smaller than those of BF and PF cells (*p* < 0.05). The compact arrangement and small size of IMF cells facilitate their even dispersion among muscle fibers, thus playing a crucial role in enhancing meat tenderness. This might be attributed to their ability to provide better lubrication and buffering during muscle contraction and relaxation, reducing the friction between muscle fibers and consequently making the meat tender in texture [18]. BF, with its loose structure and larger cells, is more suitable for storing large amounts of fat to meet the energy demands of pigs during growth and reproduction [19]. The unique morphology and distribution of PF suggest that it may serve dual functions in the physiological protection of the intestine and local energy metabolism. It potentially regulates intestinal peristalsis, absorption, and immune functions through certain bioactive substances related to gut microbiota activities [20]. Meanwhile, it also participates in the regulation of local fat metabolism and maintains a close connection with the overall energy balance [21].

In terms of fatty acid composition, the differences in fat content among various sites of pigs have a profound impact on both meat flavor and lipid metabolism [22]. Moreover, it is widely believed that unsaturated fatty acids are healthier than saturated fatty acids. Studies have found that the proportion of unsaturated fatty acids in IMF is 68%, in PF is 53%, and in BF is 57%. In the fatty acid analysis of TPs, the content of saturated fatty acids in IMF was lower than that in BF and PF, while the proportion of monounsaturated fatty acids in IMF was higher than that in the latter two. In the fatty acid analysis of TPs, C16:0 and C18:0 were the dominant saturated fatty acids. Among the monounsaturated fatty acids, oleic acid C18:1 (cis-9) had the highest content. In the adipose tissue of pork, a higher content of C16:10 and C18:0 makes the fat texture harder [23,24]. During cooking, esterification reactions occur, generating ester compounds that endow pork with its characteristic mellow aroma [25,26]. Conversely, a higher proportion of C18:1 (cis-9) imparts a more delicate, smooth, and tender texture to pork [27]. In this process, C18:1 (cis-9) is easily hydrolyzed, oxidized, and esterified, producing aldehydes, ketones, and alcohols [28,29], and these substances contribute to the unique flavor of Tibetan pork [30]. These data indicate that the composition and content of fatty acids in pork adipose tissue substantially influence the flavor and texture of pork. In the TPs, IMF contains a high level of unsaturated fatty acids, C18:1 (cis-9), which accounts for up to 42.37%. This high content of C18:1 (cis-9) is a key determinant of the tenderness and rich flavor of Tibetan pork. Therefore, the tenderness of Tibetan pork is attributed to the deposition of IMF, and the composition and content of fatty acids in intramuscular adipose tissue play a decisive role in its flavor and texture [31]. Studies have also shown that the formation of intramuscular fat is related to its unique lipid composition. An article found that lipids, such as PC-0, AcCa, and PE-0, are highly enriched in intramuscular fat, which may provide new insights for improving intramuscular fat content in future breeding [32]. The expressions of various genes play important roles in IMF deposition. Transcriptomic analysis provides a reference for elucidating the complex mechanism of the relationship between IMF, PF, and BF in TPs. One of our study’s primary aims was to clarify the roles of differentially expressed genes in various fat depots. We focused on genes enriched in signaling pathways that may be related to lipid metabolisms, such as WNT16, DDAH1, and CYP7A1. In this study, WNT16 in IMF was upregulated compared to PF and BF, while the corresponding genes DDAH1 and CYP7A1 were downregulated. WNT16 is a protein-coding gene that plays a crucial role in the WNT signaling pathway. The WNT signaling pathway is widely involved in the differentiation of mesenchymal stem cells. Research has shown that the regulation of WNT signaling can direct the differentiation of human amniotic mesenchymal stem cells in vitro toward osteogenic or adipogenic lineages. Inhibition of WNT signaling leads to downregulation of Runx2 and ALP in osteogenic differentiation, while upregulation of FABP4 in adipogenic cells [33]. Jan studied human and rodent experimental models and found that CYP7A1 mediates the conversion of cholesterol to bile acids through promoter changes regulated by thyroid hormone receptors. Menke reported that the knockout of the CYP7A1 gene in mice leads to elevated serum lipoprotein and cholesterol levels [34]. Additionally, the downregulation of MiR-96-5p can target DDAH1, promoting the proliferation, migration, and invasion of human trophoblast cells while inhibiting apoptosis [35]. Upregulation of DDAH1 may improve liver fibrosis and hepatic steatosis in obese mice by reducing the expression of S100A11 [22]. Although the detailed mechanisms of these genes in regulating fat deposition, lipid metabolism, and meat flavor are still unclear, their special expression levels in IMF suggest that they may be considered new candidate genes for intramuscular fat formation. Further research is needed to explore the specific mechanisms of these candidate genes.

The results of metabolomic analysis further enhance our understanding of the regulatory mechanisms underlying the fat characteristics of TPs. The differential metabolites, including LysoPC (17:0), LysoPC (18:0), and L-tyrosine, were screened from different fat sites of TPs. Both LysoPC (17:0) and LysoPC (18:0) belong to the class of lysophosphatidylcholines. LysoPC plays a crucial role in maintaining the structure and function of cell membranes. Changes in the content of LysoPC can affect the fluidity and permeability of cell membranes, thereby influencing the uptake of nutrients by adipocytes, the excretion of metabolites, and the transmission of intercellular signals. In the glycerophospholipid metabolic pathway, they may participate in the regulation of glycerophospholipid metabolism, impacting the processes of fatty acid synthesis, oxidation, and transport. Alterations in the content of these metabolites might trigger a series of downstream metabolic reactions, ultimately leading to differences in lipid composition and metabolic activity in different fat parts of TPs and thus exerting a significant influence on overall fat deposition and meat quality. L-tyrosine serves as the substrate for tyrosinase (TYR), an enzyme that plays a pivotal role in melanogenesis. Recent studies have highlighted that the activity of tyrosinase is not only crucial for melanin synthesis but may also influence cellular differentiation processes by modulating intracellular oxidative stress levels. In adipocytes, L-tyrosine may indirectly affect the differentiation process by regulating tyrosinase activity. This regulatory mechanism could potentially impact the balance between oxidative stress and antioxidant defense within adipocytes, thereby influencing their differentiation and function [36]. Further in-depth research on the mechanism of action of LysoPC (17:0), LysoPC (18:0), and L-tyrosine in the fat metabolism of TPs is expected to provide novel theoretical bases and potential regulatory targets for the germplasm improvement of TPs and the production of high-quality pork.

The results of the integrated transcriptomic and metabolomic analysis showed that, in the comparative group between IMF and BF, differentially expressed genes, such as WNT10B, WNT16, TSPAN6, and WNT2, along with the differential metabolite L-tyrosine, were co-enriched in the melanogenesis pathway. Previous studies have indicated that WNT10B, WNT16, TSPAN6, and WNT2 are core members of the WNT signaling pathway [37] and are also associated with the melanogenesis pathway [38]. L-tyrosine, a substrate of TYR, plays a pivotal role in melanin synthesis [39]. In contrast, no significantly enriched common metabolites or genes were detected in the comparative group between IMF and PF, which may stem from the heterogeneity of metabolic regulatory networks among different samples. Further investigation through spatiotemporal dynamic analysis or cell-type-specific studies is needed to elucidate the specific mechanisms.

Members of the WNT gene family encode secreted signaling proteins [40,41] that play a central role in the growth, metabolism, and developmental regulation of organs across various species [42]. In this study, WNT16, which exhibited the highest degree of differential expression, was selected for further investigation. WNT16, as a key member of this family, has been primarily studied in the context of skeletal biology, with its functions in fracture repair, periosteum formation, and cartilage regeneration well established [43]. However, the direct regulatory mechanisms of this gene in adipogenesis have not been fully elucidated. Studies have indicated that WNT16 is intricately linked to lipid deposition, metabolic regulation, and meat quality characteristics, and exogenous overexpression of WNT16 can promote fat formation and lipid accumulation by upregulating adipogenic markers PPARγ and C/EBPα [44,45]. Notably, PPARγ and C/EBPα, as dual-function regulators of both adipogenesis and lipid metabolism, directly control the processes of fatty acid uptake, triglyceride storage, and oxidation [46]. When the expression of these markers is suppressed, it may disrupt lipid homeostasis [47] and indirectly affect the generation of volatile flavor compounds during cooking by altering the lipid profile [48,49], thereby changing the composition of IMF—a key parameter that determines meat tenderness, juiciness, and flavor [50]. In this study, the silencing of the WNT16 gene in TPs’ preadipocytes significantly inhibited adipogenic differentiation, as evidenced by decreased expression of adipogenic marker genes PPARγ/C/EBPα, reduced lipid droplets, as quantified by Oil Red O staining, and decreased OD values. These findings not only confirm that WNT16 may participate in the maintenance of metabolic homeostasis by regulating the PPARγ/C/EBPα pathway but also suggest that it holds a central regulatory position in lipid deposition. In summary, porcine WNT16 is not only involved in skeletal development, but may also affect meat quality by influencing lipid metabolism through changes in lipogenic gene expression, which had not been shown before.

## 5. Conclusions and Prospects

In summary, this study demonstrated significant differences in adipocyte size and fatty acid composition among intramuscular, back fat, and perirenal fat depots in pigs. Integrated multi-omics analysis identified WNT16 as a differentially expressed gene correlated with the abundance of L-tyrosine, both enriched in the melanogenesis pathway. Functional assays suggested that WNT16 modulates adipogenic differentiation via regulation of PPARγ and C/EBPα. While a mechanistic link between WNT16, L-tyrosine, and melanogenesis remains to be established, our findings suggest WNT16 as a candidate regulator of depot-specific adipogenesis. Future studies, including targeted gene-editing and in vivo modeling, are needed to clarify its role in lipid metabolism and its potential application in meat quality improvement.

## Figures and Tables

**Table 1:** Primer information of qPCR.

Gene	Primer Sequences (5′-3′)	Product Size
WNT16	F: TGGTGCATTCTGTGACCAGG	111 bp
	R: AACACTCCGTCATGTTGCCT	
DDAH1	F: AGTGTCCAACGGCAACAAGA	56 bp
	R: AGCCGCGATCATCTTTGAGA	
IL6	F: GCTGCTTCTGGTGATGGCTA	76 bp
	R: TGAGGTGGCATCACCTTTGG	
PYGB	F: CAACATGGCCCACCTTTGTG	58 bp
	R: GATCCTCGCCACACCATTGA	
ADRA1B	F: TGGTCATGTACTGCCGTGTC	154 bp
	R: TTGGCCTTCGTACTGCTGAG	
CYP7A1	F: ATGAGGAGAAGGCAAACGGG	109 bp
	R: GGTTTGCTCGGAGGAACTCA	
PPARγ	F: ATTCATGACAAGGGAGTTTCTAAGG	250 bp
	R: GGAGGACTCTGGGTGGTTCA	
C/EBPα	F: GAGCCGCCCTTCACAGAG	236 bp
	R: GTCTTCGATGTCGGTCAGCA	
AdipoQ	F: TCCCTAACATGCCCATTCGC	240 bp
	R: CAAGTAGACCGTGACGTGGA	
Actin	F: GGACTTCGAGCAGGAGATGG	233 bp
	R: GCACCGTGTTGGCGTAGAGG	

**Table 2:** siRNA sequence designed for WNT16.

Plasmid Name	siRNA Sequence
si-WNT16-5132	5′-GCACCAAGGAAACAGCAUUTT-3′ 5′-AAUGCUGUUUCCUUGGUGCTT-3′
si-WNT16-663	5′-GGGCUGCUCUGAUGAUGUUTT-3′ 5′-AACAUCAUCAGAGCAGCCCTT-3′
si-WNT16-749	5′-GCAAAGUACUGUUAGCAAUTT-3′ 5′-AUUGCUAACAGUACUUUGCTT-3′
Negative control	5′-UUCUCCGAACGUGUCACGUTT-3′ 5′-ACGUGACACGUUCGGAGAATT-3′

**Table 3:** Fatty acid content of different parts of TPs.

Fatty Acid Types	BF of Content (g/100 g)	PF of Content (g/100 g)	IMF of Content (g/100 g)
C8:0	0.009 ± 0.001	0.007 ± 0.001	0.008 ± 0.001
C10:0	0.073 ± 0.003 ^b^	0.095 ± 0.004 ^a^	0.087 ± 0.003 ^a^
C12:0	0.080 ± 0.006	0.100 ± 0.008	0.094 ± 0.006
C14:0	1.410 ± 0.087	1.577 ± 0.073	1.527 ± 0.089
C14:1 (cis-9)	0.024 ± 0.005	0.015 ± 0.003	0.032 ± 0.006
C15:0	0.054 ± 0.006	0.063 ± 0.013	0.043 ± 0.005
C16:0	25.133 ± 0.649	27.067 ± 0.700	25.933 ± 0.726
C16:1 (cis-9)	2.860 ± 0.460 ^ab^	1.840 ± 0.269 ^b^	3.580 ± 0.580 ^a^
C17:0	0.246 ± 0.025 ^ab^	0.333 ± 0.049 ^a^	0.190 ± 0.214 ^b^
C18:0	11.467 ± 0.533 ^b^	17.233 ± 1.167 ^a^	11.067 ± 0.448 ^b^
C18:1 (cis-9)	33.267 ± 6.763	32.200 ± 1.069	42.366 ± 0.696
C18:2 (all-cis-9,12)	15.267 ± 0.120 ^a^	16.333 ± 1.369 ^a^	11.967 ± 0.176 ^b^
C18:3 (all-cis-6,9,12)	0.030 ± 0.001 ^ab^	0.038 ± 0.004 ^a^	0.028 ± 0.001 ^b^
C18:3 (all-cis-9,12,15)	0.654 ± 0.013 ^ab^	0.728 ± 0.063 ^a^	0.526 ± 0.001 ^b^
C20:0	0.272 ± 0.010 ^ab^	0.297 ± 0.009 ^a^	0.259 ± 0.010 ^b^
C20:1 (cis-11)	1.086 ± 0.043 ^a^	0.760 ± 0.015 ^b^	1.054 ± 0.043 ^a^
C20:2 (all-cis-11,14)	0.860 ± 0.044 ^a^	0.706 ± 0.034 ^b^	0.647 ± 0.036 ^b^
C20:3 (all-cis-11,14,17)	0.146 ± 0.009 ^a^	0.108 ± 0.006 ^b^	0.107 ± 0.005 ^b^
C20:3 (all-cis-8,11,14)	0.130 ± 0.005 ^a^	0.129 ± 0.006 ^a^	0.108 ± 0.003 ^b^
C20:4 (all-cis-5,8,11,14)	0.267 ± 0.008 ^ab^	0.313 ± 0.023 ^a^	0.254 ± 0.007 ^b^
C20:5 (all-cis-5,8,11,14,17)	0.006 ± 0.001	0.013 ± 0.005	0.004 ± 0.002
C22:0	0.016 ± 0.001	0.017 ± 0.003	0.016 ± 0.001
C22:1 (cis-13)	0.019 ± 0.001	0.015 ± 0.002	0.019 ± 0.001
C22:2 (all-cis-13,16)	0.014 ± 0.001	0.012 ± 0.001	0.012 ± 0.000
C22:6 (all-cis-4,7,10,13,16,19)	0.014 ± 0.000 ^b^	0.022 ± 0.001 ^a^	0.013 ± 0.002 ^b^

Values in the same row with different letter superscripts mean a significant difference (*p* < 0.05).

## Data Availability

The raw datasets used and analyzed during the current study are available from the corresponding author upon reasonable request.
